# Why is asymptomatic bacteriuria overtreated?: A tertiary care institutional survey of resident physicians

**DOI:** 10.1186/s12879-015-1044-3

**Published:** 2015-07-26

**Authors:** Myung Jin Lee, Moonsuk Kim, Nak-Hyun Kim, Chung-Jong Kim, Kyoung-Ho Song, Pyoeng Gyun Choe, Wan Beom Park, Ji Hwan Bang, Eu Suk Kim, Sang Won Park, Nam Joong Kim, Myoung-don Oh, Hong Bin Kim

**Affiliations:** Department of Internal Medicine, Seoul National University College of Medicine, 101 Daehak-ro, Jongno-gu, Seoul 110-744 Republic of Korea; Department of Internal Medicine, Seoul National University Bundang Hospital, 173 Gumi-ro, Bundang-gu, Seongnam, Gyeonggi-do 463-707 Republic of Korea

**Keywords:** Asymptomatic bacteriuria, Urinary tract infection, Antibiotics, Survey, Physicians

## Abstract

**Background:**

Asymptomatic bacteriuria (ABU) is common and often leads to unnecessary antimicrobial use. Reducing antibiotic overuse for ABU is therefore an important issue for antimicrobial stewardship. We performed this study to investigate the appropriateness of ABU management and to evaluate physicians’ knowledge and practice regarding ABU.

**Methods:**

We reviewed all urine cultures of ≥10^5^ cfu/mL of bacteria among inpatients in a 900-bed hospital in 2011. Each episode of bacteriuria was classified into ABU or urinary tract infection (UTI). ABU was defined as a positive urine culture (≥10^5^ cfu/mL) without symptoms or signs suggesting UTI. In October 2012 a cross-sectional survey of resident physicians was undertaken using an anonymous, self-administered questionnaire.

**Results:**

We identified 219 ABU cases among 1167 positive urine cultures, of which 70 (32.0 %) were inappropriately treated. Female gender, old age, pyuria, hematuria, and positive nitrite on urinalysis were associated with inappropriate ABU treatment in a multivariate analysis (*P* < 0.05).

The response rate to the survey was 74.2 % (95/128). The mean knowledge score was 37.3 %, and 33.7 % of respondents were able to distinguish ABU from UTI, but less than half knew the indications for treating ABU. Even after ABU was correctly diagnosed, concerns about postoperative infections (38.6 %), UTI (9.1 %), and abnormal urinalysis (29.5 %) prevented proper management. About half of the respondents reported to prescribing antibiotics for ABU despite knowing they were not indicated.

**Conclusions:**

About one third of ABUs were inappropriately managed. Lack of knowledge and discrepancies between knowledge and practice, contributed to antimicrobial overuse for ABU. Our findings highlight the importance of developing interventions, including education, audit and feedback, to tackle the problem of inappropriate treatment of ABU.

**Electronic supplementary material:**

The online version of this article (doi:10.1186/s12879-015-1044-3) contains supplementary material, which is available to authorized users.

## Background

Urinary tract infection (UTI) is the most common nosocomial infection, accounting for about 40 % of healthcare-associated infections [1]. However, asymptomatic bacteriuria (ABU) is also prevalent, and often misdiagnosed as UTI leading to inappropriate antimicrobial use [2–6]. Antibiotic overuse has several adverse effects, including the emergence of multidrug resistant organisms, adverse drug reactions, *Clostridium difficile* infection, and increased costs of health care [7–9].

Although current guidelines suggest screening for ABU and treating it in specific circumstances such as during pregnancy or before invasive urologic procedures, antibiotic overuse for ABU seems to be overwhelming in clinical practice, as supported by several studies reporting that 20–80 % of cases of ABU are inappropriately treated [4–6, 10, 11]. Studies have shown that patients’ advanced age, the causative organism, and pyuria are factors associated with unnecessary ABU treatment [4, 5]. Physicians’ lack of knowledge and misperceptions also contribute to the misuse of antibiotics [12, 13].

Reducing unnecessary antibiotic use for ABU is a high-yield area in the antibiotic stewardship program, considering the large amounts of antibiotics used for UTI in hospitalized patients and the frequent misdiagnosis of ABU as UTI [14]. It is also an important component of performance measures and quality improvement in patient care [15, 16].

In order to optimize antibiotic use and improve guideline-compliant practices, it is crucial to assess prevailing practice and identify realistic targets. However, the issues of bacteriuria management and its associated factors in Korea have not been investigated. Therefore, we performed this study to assess the appropriateness of ABU management and to evaluate clinical and physician-related factors including knowledge, perception, and practice regarding ABU. This information should be valuable for designing further interventions to enhance the proper management of bacteriuria.

## Methods

### Case selection and data collection

We retrospectively reviewed all the positive urine cultures obtained from the adult inpatients, from January to December in 2011, in a 900-bed university-affiliated tertiary care hospital in Korea. Positive urine cultures were defined as the presence of bacteria or yeast ≥10^5^ cfu/mL, because only cultures yielding ≥10^5^ cfu/mL of microorganisms are routinely reported in the hospital unless there is a request for reporting low-count bacteriuria. Since the unit of analysis in our study was a single episode of bacteriuria, repeated bacteriuria examinations of a single patient were counted as separate events. Urine cultures made among patients in the emergency room or outpatient departments were not reviewed. Similarly excluded were urine cultures from 1) pregnant women, 2) patients undergoing invasive urologic procedures, and 3) patients with concomitant infections receiving antibiotics for infections other than UTI at the time of urine collection. Urine cultures made within seven days of a previous bacteriuric event were excluded as these were likely to involve the same episode of bacteriuria. Urine cultures performed within 48 h of admission were also excluded as these would represent episodes of bacteriuria with onset before admission and therefore hard to assess. However, since we did not try to distinguish between catheter-associated bacteriuria and non-catheter- associated, patients with urine catheters were included.

We applied standardized criteria for diagnosing ABU and UTI, modified from the Centers for Disease Control and Prevention surveillance criteria as well as the 2005 Infectious Disease Society of America (IDSA) guidelines [10, 17]. UTI was defined as the presence of bacteriuria or funguria ≥10^5^ cfu/mL along with at least one of the following symptoms and signs with no other recognized cause: fever (temperature ≥37.8 °C), dysuria, urgency, frequency, suprapubic tenderness, and costovertebral angle pain or tenderness. ABU was defined as bacteriuria or funguria ≥10^5^ cfu/mL, without any symptoms or signs suggesting UTI. We did not confine ABU or UTI to bacteriuria involving no more than 2 species of microorganisms, as we were not concerned to distinguish between true bacteriuria and contamination. Antibiotic use for ABU, except for pregnant patients and those undergoing traumatic urologic procedures in which mucosal bleeding is anticipated, is regarded as inappropriate, according to the IDSA guidelines [10]. Two infectious diseases (ID) specialists independently reviewed the medical records of patients with positive urine cultures and classified each bacteriuric episode as ABU or UTI and assessed the appropriateness of management. If the two opinions differed, another ID specialist was consulted and the final decision was made by majority.

In order to identify clinical factors associated with antibiotic use for ABU, we collected data on patient demographics, the admitting department, underlying disease including diabetes mellitus, chronic kidney disease, spinal cord injury and solid tumor, as well as indwelling urinary catheter, organisms found in urine culture, urinalysis and other laboratory findings, body temperature and antibiotic use.

Our study was approved by the institutional review board of Seoul National University Bundang Hospital.

### Survey of physicians regarding ABU

We conducted a cross-sectional survey over two weeks in October 2012, using an anonymous, self-administered, paper-based questionnaire to evaluate physician-related factors affecting ABU management. All resident physicians were eligible for the survey, except pediatricians and those affiliated with departments without hospitalized patients, namely radiology, pathology, anesthesiology, nuclear medicine, laboratory medicine, and radiation oncology. Resident physician was defined as a person enrolled in the official 4-year-long resident training program after finishing 1 year of internship. Although senior doctors often influence junior doctors when the latter are making clinical decisions, they were not surveyed because we targeted those physicians who were primarily in charge of issuing orders for and managing inpatients. One researcher contacted the resident physicians in each department to ask them to participate on a voluntary basis, and then retrieved the questionnaires with informed consents two weeks later. No incentives for participation were provided.

The questionnaire was based upon prior research and revised after review by infectious disease physicians and professionals in our institution [12, 13]. Consisting of 12 questions about bacteriuria and 4 demographic questions, the questionnaire was divided into three dimensions; 1) assessment of knowledge using seven brief clinical vignettes, 2) evaluation of perceptions, 3) understanding of actual practice. In the knowledge assessment part, respondents were provided different virtual clinical cases which we had developed for the survey and were asked to select one of three options concerning the diagnosis (ABU/ UTI/ uncertain) and management (would prescribe antibiotics/ would not prescribe antibiotics/ not sure) for each vignette. The knowledge score was measured by the proportion of correct answers. The perception part consisted of questions focused on the clinical approach to bacteriuria; 1) reasons for ordering urine cultures, 2) reactions to abnormal urinalysis findings, 3) factors to consider in diagnosing UTI. The practice part included questions about adhering to the guidelines in reality and perceived reasons for incorrect practices. The demographic questions consisted of gender, age, training level, and affiliated department. The questionnaire is presented in more detail in Additional file 1.

### Statistical analysis

Fisher’s exact test or the chi-square test was used to compare categorical variables, and the Mann–Whitney *U* test for comparison of continuous data. Variables with a *P* value < 0.1 in univariate analysis were included in a multivariate analysis using logistic regression to identify factors associated with inappropriate treatment of ABU. All *P* values were two-tailed, and statistical significance was accepted at a *P* value < 0.05*.* PASW for Windows (version 18 software package; SPSS Inc., Chicago, IL, USA) was used for the analysis.

## Results

### Inappropriate antibiotic use in ABU

**Fig. 1:**
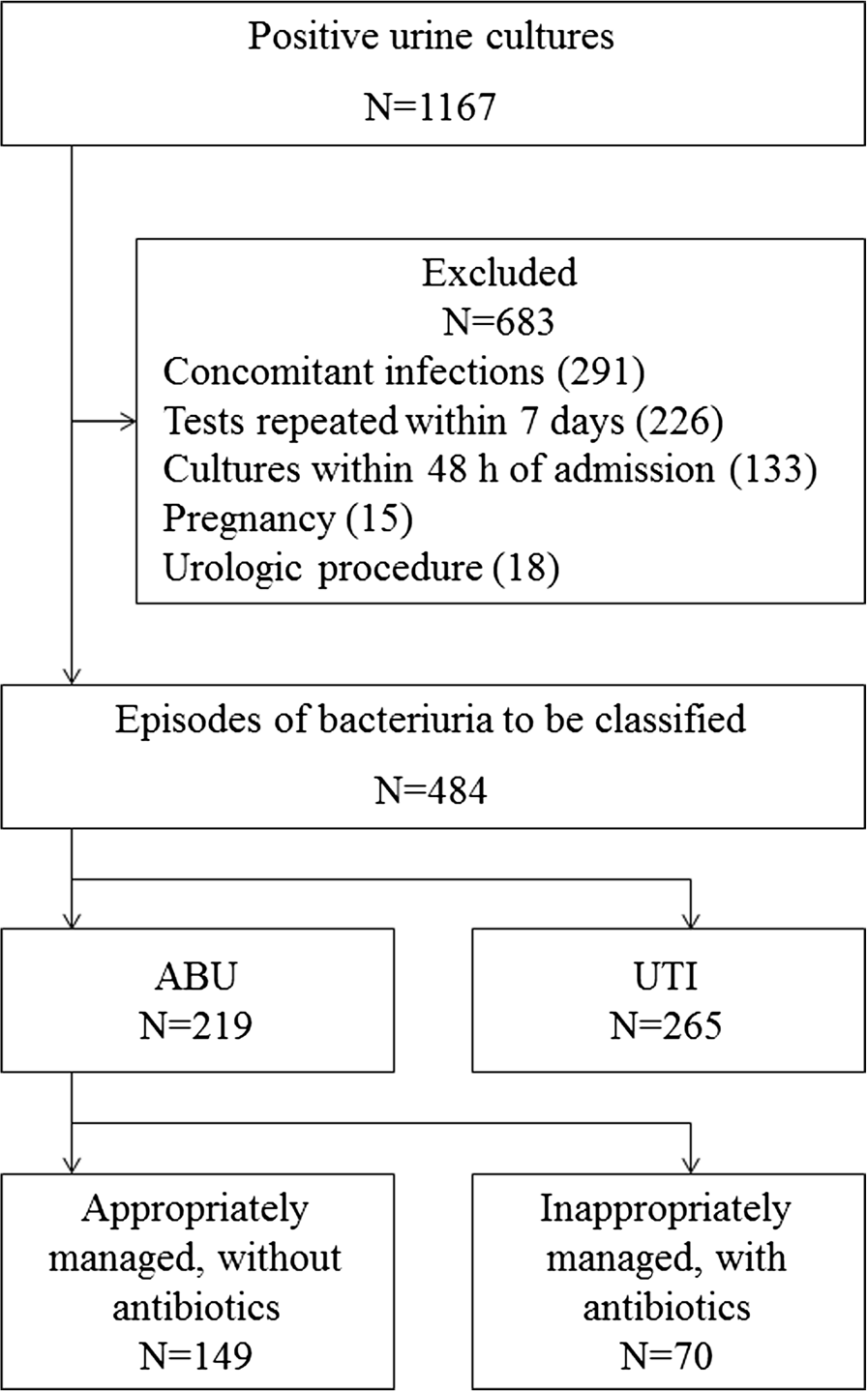
Flow chart of case enrollment and classification

A total of 6,269 urine cultures were performed among adult inpatients during the study period, of which 1,529 grew microorganisms. Among these we identified 1,167 episodes of positive urine cultures that grew ≥10^5^ cfu/mL, and another 362 episodes with lower counts. As shown in Fig. 1, 683 episodes of bacteriuria were excluded. Among the remaining 484 cases of bacteriuria, 219 cases were classified as ABU. These 219 ABU episodes involved 183 patients, of which 149 (68.0 %) were appropriately managed whereas 70 (32.0 %) were inappropriately treated with antibiotics.

A comparison of the two groups is shown in Table 1. The median age was higher in the inappropriately-managed group (*P* = 0.014) and female gender, pyuria, hematuria, bacteriuria, positive nitrite on urinalysis, and gram negative organism on urine culture were all related to unnecessary antibiotic use in univariate analysis. In multivariate analysis, patient age, female gender, pyuria, hematuria, and positive nitrite on urinalysis were significantly associated with inappropriate management of ABU (*P* < 0.05, by logistic regression).

**Table 1:** Comparison between patients with appropriately-managed and inappropriately-managed ABU^a^

			Univariate analysis		Multivariate analysis	
Variables	Appropriately- managed ABU	Inappropriately- managed ABU	OR	*P* value	aOR	*P* value
	(*n* = 149)	(*n* = 70)	(95 % CI)		(95 % CI)	
Age (year) (median, IQR)	69 (57–77)	73 (69–80)		0.014	1.03 (1.00-1.06)	0.040
Body temperature ≥37.8 °C	17 (11.4)	2 (2.9)	0.23 (0.05-1.02)	0.036	0.34 (0.06-2.07)	0.243
Female gender	100 (67.1)	61 (87.1)	3.32 (1.52-7.24)	0.002	3.38 (1.19-9.66)	0.023
Admission to surgical department^b^	51 (34.2)	25 (35.7)	1.07 (0.59-1.94)	0.829		
Urinary catheterization	73 (49.0)	40 (57.1)	1.39 (0.78-2.46)	0.260		
Stay in ICU	11 (7.4)	6 (8.6)	1.18 (0.42-3.32)	0.759		
Underlying diseases						
Diabetes mellitus	54 (36.2)	27 (38.6)	1.11 (0.62-1.98)	0.739		
Chronic kidney disease	17 (11.4)	2 (2.9)	0.23 (0.05-1.02)	0.036	0.23 (0.04-1.26)	0.090
Spinal cord injury	6 (4.0)	5 (7.1)	1.83 (0.54-6.23)	0.325		
Cancer	23 (15.4)	9 (12.9)	0.81 (0.78-2.46)	0.614		
Leukocytosis (WBC >10^4^/mm^3^)	36 (24.2)	24 (34.8)	1.67 (0.90-3.12)	0.102		
Elevated CRP (>0.5 mg/dL)	100 (67.1)	40 (57.1)	1.00 (0.47-2.15)	1.0		
Urinalysis						
Pyuria	105 (70.5)	65 (92.9)	5.45 (2.05-14.45)	<0.001	4.07 (1.30-12.71)	0.016
Hematuria	44 (29.5)	37 (52.9)	2.68 (1.49-4.81)	0.001	4.48 (2.09-9.58)	<0.001
Bacteriuria	118 (79.2)	66 (94.3)	4.34 (1.47-12.82)	0.004	2.39 (0.63-9.10)	0.200
Nitrite positivity	51 (34.2)	52 (74.3)	5.55 (2.95-10.46)	<0.001	2.21 (1.01-4.85)	0.048
Urine culture						
Gram negative bacteria	85 (57.0)	63 (90.0)	6.78 (2.91-15.78)	<0.001	2.06 (0.66-6.39)	0.213

Note. Data are no. (%) of patients

^a^ Variables with a *P* value < 0.1 in the univariate analysis were included in the multivariate logistic regression analysis

^b^ Includes General Surgery, Neurosurgery, Orthopedic Surgery, Thoracic Surgery, Urology, Otorhinolaryngology, Obstetrics and Gynecology, and Ophthalmology

Abbreviation: *ABU* asymptomatic bacteriuria, *CI* confidence interval, *OR* odds ratio, *aOR* adjusted odds ratio, *IQR* interquartile range, *ICU* intensive care unit, *WBC* white blood cell, *CRP* C-reactive protein

The patients who were inappropriately treated were prescribed antibiotics for a median of six days (interquartile range 4 – 8 days). Ciprofloxacin (67.1 %), third-generation cephalosporins (25.7 %), and ertapenem (4.3 %) were the most frequently used antibiotics.

### Survey of resident physicians

Of 128 residents, 95 (50 from medical departments and 45 from surgical) completed the questionnaire, giving an overall response rate of 74.2 %. Of the respondents, 66.3 % were male, and the median age was 29 years (interquartile range, 27–31 years). In terms of stage of training, 30 (31.6 %) were in their first year, 24 (25.3 %) in their second, 21 (22.1 %) in their third, and 20 (21.1 %) in their fourth.

The mean frequency (± SD) of correct responses to the seven clinical vignettes was 37.3 % (±26.7 %), and there was a significant difference between the specialties that were being followed; 44.0 % (±30.0 %) for those in medical departments, 29.8 % (±20.4 %) for those in surgical departments (*P* = 0.008). No significant association was found between the knowledge score and stage of training (*P* = 0.92). In general, the respondents showed poor recognition of ABU (33.7 %) and also had a limited understanding of the indications for antibiotic use (20.0 %), judging from the responses to the clinical vignettes involving ABU (Table 2). Nevertheless, most of the respondents were aware that antibiotics were not indicated for ABU, as shown by the > 90 % correct responses concerning treatment in the vignettes describing ABU for which no therapy was needed (Table 2).

**Table 2:** Summary of clinical vignettes with the corresponding proportions of correct responses

Summary of clinical vignettes		Diagnosis	Treatment
	Expected responses to diagnosis and management	Correct response /total responses (%)	Correct response/correct diagnosis (%)
1. A 50-year-old man with hypertension was seen for annual physical exam, with no urinary symptoms. Routine UA showed pyuria; UC grew ≥10^5^/ml of *Escherichia coli.*	ABU, no treatment is needed	31/95 (32.6)	31/31 (100)
2. A 70-year-old woman with recurrent UTI history admitted due to trauma, without urinary symptoms. UA showed pyuria; UC grew ≥10^5^/ml of *Escherichia coli.*	ABU, no treatment is needed	42/95 (44.2)	39/42 (92.9)
3. A 68-year-old man with an indwelling foley catheter had cloudy urine, without urinary symptoms or signs of infection. UA showed pyuria; UC grew ≥10^5^/ml of *Klebsiella pneumoniae.*	ABU, no treatment is needed	19/95 (20.0)	18/19 (94.7)
4. A 82-year-old woman without urinary symptoms was seen preoperatively before total knee arthroplasty. A preoperative UC grew ≥10^5^/ml of *Klebsiella pneumoniae.*	ABU, no treatment is needed	37/95 (38.9)	34/37 (91.9)
5. A pregnant woman at 12 weeks’ gestation without urinary symptoms presented with pyuria, nitrite positivity on UA. UC grew ≥10^5^/ml of *Escherichia coli*.	ABU, indicated for antibiotic therapy	29/95 (30.5)	17/29 (58.6)
6. A 75-year-old man was about to undergo transurethral resection of the prostate. A preoperative UC grew ≥10^5^/ml of *Klebsiella pneumoniae.*	ABU, indicated for antibiotic therapy	34/95 (35.8)	21/34 (61.8)
7. A 68-year-old woman admitted to the ICU with altered mentality due to drug intoxication developed SIRS. She had an indwelling Foley catheter. UC grew ≥10^5^/ml of *Escherichia coli.* No other suspected infection focus was found.	UTI, indicated for antibiotic therapy	89/95 (93.7)	88/89 (98.9)

Note. Clinical vignettes provided on the questionnaire are virtual cases developed for the purpose of surveying resident physicians

Abbreviation: *UA* urinalysis, *UC* urine culture, *ABU* asymptomatic bacteriuria, *UTI* urinary tract infection, *ICU* intensive care unit, *SIRS* systemic inflammatory response syndrome

All of the respondents answered that they would not initiate antibiotics simply for abnormal urinalysis results, but would try to make a reasoned decision concerning antibiotic prescription based on symptoms or signs suggesting UTI, repeated urinalysis or urine culture results, and other laboratory findings. However, they tended to order urine cultures unnecessarily in asymptomatic patients for the following reasons: to identify occult infection even in cases without compatible symptoms or signs; reflexively in response to abnormal urinalysis results; and for preoperative surveillance.

About half of the respondents (46.3 %) confessed to prescribing antibiotics for ABU despite knowing they were not indicated. There was no association between self-reported discordant practice and mean knowledge scores (*P* = 0.18) or stage of training (*P* = 0.41). Self-perceived reasons for initiating antibiotics despite a diagnosis of ABU were: concern about ABU leading to postoperative infectious complications (38.6 %) or symptomatic UTI (9.1 %); elevated inflammatory markers (36.4 %) and abnormal urinalysis results (29.5 %). Concern about postoperative infection was higher among surgical residents than non-surgical residents (38.6 % vs 0 %, *P* < 0.001).

## Discussion

We set out to determine why antibiotics are overprescribed for ABU by evaluating both patient and physician factors associated with inappropriate treatment of ABU. About one-third of ABUs were overtreated against guidelines, in agreement with earlier studies [4–6]. Female gender, old age, and pyuria, hematuria, and positive nitrite on urinalysis were the patient clinical factors contributing to antibiotic overuse, while lack of knowledge, misperception of the clinical implications of bacteriuria, and discordance between knowledge and practice were the physician-related factors. One strength of our study is that we have attempted to identify such patient- and physician-related factors, as most previous studies focused on clinical factors or physicians’ knowledge and attitudes alone [4–6, 12, 13]. Furthermore, our data are valuable because of the high response rate to the survey (74.2 %) compared to previous studies and the participation of physicians from a variety of departments, all of which can be helpful for developing effective antimicrobial stewardship programs and interventions [12, 13].

Pyuria was the most important clinical factor related to inappropriate antibiotic treatment of ABU. However, pyuria itself does not distinguish UTI from ABU nor does it indicate antibiotic use, being commonly found in patients with ABU, ranging from 30 % in young women to 90 % in the elderly or hemodialysis patients [3, 10, 18, 19]. This finding agrees with the survey result that 29.5 % of respondents prescribed antibiotics for ABU in actual practice for abnormal urinalysis results, regardless of the presence or absence of urinary symptoms. We assume that physicians find it difficult to neglect pyuria despite the absence of urinary symptoms, or that they find it difficult to distinguish UTI from ABU with pyuria. Likewise, physicians seemed to have serious concerns about the possibility of UTI developing in the elderly, and about women with ABU, even though ABU is prevalent in these groups. These results are consistent with earlier studies which reported pyuria, old age, and type of organism as factors promoting antibiotic overuse for ABU [4, 5]. Therefore, education about the differential diagnosis of pyuria and the interpretation of urinalysis is required to improve the understanding and management of bacteriuria.

Our survey demonstrated an overall paucity of knowledge regarding bacteriuria and considerable knowledge vs. gaps in practice among physicians. This agrees with previous surveys reporting resident physicians’ (48 %) and healthcare providers’ (56-71 %) poor knowledge regarding urine testing and guidelines about ABU, although the mean level of knowledge was lower in our survey (37.3 %) [13, 20]. For the clinical vignettes describing ABU where antibiotics were not indicated, about one third of the respondents correctly chose both the diagnosis and management. Most of the physicians who were able to diagnose ABU said antibiotics should not be used for such cases, whereas those who could not identify ABU opted to initiate antibiotics. However, for the vignettes describing ABU that required treatment (pregnancy, prior to transurethral resection of prostate), only half of the respondents who made a correct diagnosis were aware of the need for antibiotics. Therefore, we suppose that the 32.0 % rate of inappropriately managed ABU in the retrospective chart review is mainly due to physicians’ misdiagnosis of ABU as UTI, and may be influenced by various clinical factors that confuse physicians about the diagnosis. To improve the current situation, clinical scenario-based education, not just dependence on guidelines or textbooks, and specific trainee-centered education may be helpful considering the inter-specialty difference in knowledge demonstrated by the survey.

From the survey, we could see that urine cultures were often ordered regardless of the probability of UTI for various reasons mostly associated with misunderstanding of bacteriuria. This effect was also evident in our review of bacteriuria cases, as the indications for urine culture were often not clarified on the medical records. Concern about ABU being a risk factor for postoperative infection or symptomatic UTI lead physicians to check for the presence of bacteriuria, and thereafter to unnecessary antimicrobial use to eradicate ABU. Although concern about postoperative infection originating from ABU is prevalent among surgeons, especially in prosthesis implant surgery, evidence is mounting that neither routine urine culture in asymptomatic patients nor preoperative eradication of ABU is useful [21, 22]. Although this remains controversial, we generally do not recommend treating ABU before hip arthroplasty in the study hospital. In addition, a recent study has shown that suppressing reports of urine culture results ordered for non-catheterized patients was effective in reducing reflexive antibiotic use for ABU [23]. Therefore, we suggest that reducing unindicated urine cultures may be the first step to decreasing antibiotic misuse and to improving physicians’ recognition of the difference between UTI and ABU.

We found considerable discrepancies between knowledge, perception and clinical practice, which made the physician factor significant in relation to the inappropriate treatment of ABU. Nearly half of the respondents acknowledged that their own actual practice was at variance with their knowledge. Although the majority of the respondents agreed that patients’ symptoms or signs were important for the diagnosis of UTI and none opted to initiate antibiotics reflexively for abnormal urinalysis results, the lack of documented explanations of the rationale for antibiotic use for bacteriuria indicates a prevalent discrepancy between practice and perception. Such attitudes of physicians were alluded to in a previous survey of the management of bacteriuria by intensive care unit clinicians [12]. Practice-based audit, and feedback to help physician detect the errors of their practices, as well as fundamental educational efforts are of critical importance in overcoming this gap. Further studies are needed to explore the drivers of physician practice that contradicts their knowledge.

Our partly retrospective, partly prospective study has a few limitations. First, there may have been some misclassification of episodes of bacteriuria because of the retrospective part of the study. However, to minimize possible biases incurred from a retrospective review, three ID specialists independently participated in the classification and evaluation of the appropriateness of antibiotic use. Second, our study was conducted in an acute care hospital, so our result may not be generalizable to other situations such as long-term care facilities where ABU is much more prevalent. Third, as we surveyed physicians in the year after the care was provided (2012), some of the residents were not the same as those who actually cared for the episodes of bacteriuria in 2011. However, we believe that the inappropriate antibiotic use data can still be reasonably linked and correlated with the survey considering that the majority of the residents surveyed did practice in 2011.

## Conclusions

About one third of ABUs are overtreated and female gender, old age, and abnormal urinalysis results are associated with such inappropriate treatment. Physician-related factors including knowledge deficit, cognitive biases, and discrepancies between perception and practice are factors that could be modified to reduce antibiotic overuse for ABU. Therefore, interventions addressing both education to improve knowledge, and audit and feedback to overcome the barriers to following guidelines, are warranted.

## Additional file

Additional file 1:
**Questionnaire for resident physicians. **(DOCX 20 kb)
