# Supplementary material for: Why is asymptomatic bacteriuria overtreated?: A tertiary care institutional survey of resident physicians
Source: BMC Infect Dis. 2015 Jul 26;15:289. doi: 10.1186/s12879-015-1044-3 (PMC4514993; doi:10.1186/s12879-015-1044-3)
Supplement: Additional file 1: — Questionnaire for resident physicians. (DOCX 20 kb) [file 12879_2015_1044_MOESM1_ESM.docx]

**SUPPLEMENTARY DATA**

**Questionnaire for resident physicians**

**♣** Please read the following clinical vignettes and indicate your decisions about diagnosis and treatment.

1. A 50-year-old man with hypertension was seen for his annual physical exam, and had no urinary symptoms. Routine UA showed pyuria; UC grew ≥ 10^5^/ml of *Escherichia coli.*

1) What is your diagnosis?

□ Asymptomatic bacteriuria □ Urinary tract infection □ Uncertain

2) Would you prescribe antibiotics?

□ Yes □ No □ Not sure

2. A 70-year-old woman with a history of recurrent UTI was admitted due to trauma, without urinary symptoms. UA showed pyuria; UC grew ≥ 10^5^/ml of *Escherichia coli*

1) What is your diagnosis?

□ Asymptomatic bacteriuria □ Urinary tract infection □ Uncertain

2) Would you prescribe antibiotics?

□ Yes □ No □ Not sure

3. A 68-year-old man with an indwelling Foley catheter had cloudy urine, without urinary symptoms or signs of infection. UA showed pyuria; UC grew ≥ 10^5^/ml of *Klebsiella pneumoniae.*

1) What is your diagnosis?

□ Asymptomatic bacteriuria □ Urinary tract infection □ Uncertain

2) Would you prescribe antibiotics?

□ Yes □ No □ Not sure

4. An 82-year-old woman without urinary symptoms was seen preoperatively before total knee arthroplasty. A preoperative UC grew ≥ 10^5^/ml of *Klebsiella pneumoniae.*

1) What is your diagnosis?

□ Asymptomatic bacteriuria □ Urinary tract infection □ Uncertain

2) Would you prescribe antibiotics?

□ Yes □ No □ Not sure

5. A pregnant woman at 12 weeks of gestation without urinary symptoms presented with pyuria, nitrite positivity on UA. UC grew ≥ 10^5^/ml of *Escherichia coli*.

1) What is your diagnosis?

□ Asymptomatic bacteriuria □ Urinary tract infection □ Uncertain

2) Would you prescribe antibiotics?

□ Yes □ No □ Not sure

6. A 75-year-old man was about to undergo transurethral resection of the prostate. A preoperative UC grew ≥ 10^5^/ml of *Klebsiella pneumoniae.*

1) What is your diagnosis?

□ Asymptomatic bacteriuria □ Urinary tract infection □ Uncertain

2) Would you prescribe antibiotics?

□ Yes □ No □ Not sure

7. A 68-year-old woman admitted to the ICU with altered mentality due to drug intoxication developed SIRS. She had an indwelling Foley catheter. UC grew ≥ 10^5^/ml of *Escherichia coli.* No other suspected infection focus was found.

1) What is your diagnosis?

□ Asymptomatic bacteriuria □ Urinary tract infection □ Uncertain

2) Would you prescribe antibiotics?

□ Yes □ No □ Not sure

**♣** Please read the following questions and indicate your opinion (multiple choices are allowed except for question #11).

8. What would you do next for a patient without any lower urinary tract symptoms but with pyuria and bacteriuria in his/her routine urinalysis?

□ Ask the patient again about the existence of urinary symptoms and repeat the physical exams

□ Repeat the urinalysis in view of the possibility of contamination or laboratory error

□ Order urine culture immediately

□ Check for leukocytosis or elevated inflammatory markers

□ Prescribe empiric antibiotics for urinary tract infection

9. If you have ever ordered urine cultures for patients without urinary symptoms, what was the reason for doing so?

□ I order urinalysis with urine culture for every patient on admission or for those who are undergoing surgery, as a routine screening

□ I order urine culture for patients with abnormalities in routinely performed urinalysis, such as pyruia, hematuria, or positive nitrite

□ I order urine culture as one of a routine panel of tests for patients suspected of having an infectious disease, not only when urinary tract infection is strongly suspected

□ I order urine culture routinely before prescribing antibiotics even if it is not for urinary tract infection

□ I order urine culture for patients with indwelling urinary catheter as a routine screening

10. What would you take into account in diagnosing urinary tract infection when a patient gives a positive urine culture (≥ 10^5^ cfu/mL of microorganism)?

□ Patient’s urinary symptoms or signs including dysuria, urgency, frequency, suprapubic pain or tenderness or fever

□ Blood test results including peripheral white blood cell counts, erythrocyte sedimentation rate, or level of C-reactive protein (CRP)

□ Presence of any abnormalities in the urinalysis (e.g. pyuria, hematuria, positive nitrite)

□ Existence of an indwelling urinary catheter

11. Have you ever prescribed an antimicrobial agent to a patient with asymptomatic bacteriuria despite being aware that it is not indicated?

□ Yes □ No

12. If you answered ‘yes’ to question #11, please indicate the reason of prescribing the antimicrobial agent.

□ Because of the patient’s abnormal urinalysis

□ Because of the patients’ laboratory findings with elevated CRP or leukocytosis

□ In order to prevent surgical complications for a patient undergoing surgery

□ In order to prevent asymptomatic bacteriuria from developing into symptomatic urinary tract infection

□ Because of the patient’s past history of urinary tract infection

□ Because the patient was immunocompromised

□ Because of a senior doctor’s pressure to prescribe antibiotics against my judgment

**♣** Please answer the following questions regarding your demographic status

13. What is your gender? □ Male □ Female

14. What is your age? ( ) years old

15. What is your specialty in training?

□ Internal medicine □ Neurology □ Rehabilitation medicine

□ Psychiatrics □ General surgery □ Neurosurgery

□ Orthopedic surgery □ Plastic surgery □ Thoracic surgery

□ Urology □ Otorhinolaryngology □ Obstetrics and Gynecology

□ Ophthalmology

16. What is your level of residency?

□ 1^st^ year of residency □ 2^nd^ year of residency

□ 3^rd^ year of residency □ 4^th^ year of residency
